# Application and challenges of a metaverse in medicine

**DOI:** 10.3389/frobt.2023.1291199

**Published:** 2023-12-11

**Authors:** Yingshu Wang, Congcong Li, Lai Qu, Hongfei Cai, Yingying Ge

**Affiliations:** ^1^ College of Art, Changchun University of Technology, Changchun, Jilin, China; ^2^ Department of Thoracic Surgery, The First Hospital of Jilin University, Changchun, Jilin, China; ^3^ Department of Critical Medicine, The First Hospital of Jilin University, Changchun, Jilin, China

**Keywords:** metaverse, medicine, virtual reality, augmented reality, challenge

## Abstract

Metaverse has been confirmed as a relatively amorphous concept of innovation, which refers to technological advancement. Metaverse, i.e., a coalition between reality world and virtual world, has created significant significance and convenience in education, communication, economy, etc. The COVID-19 outbreak has stimulated the growth of metaverse applications in medicine. The above-mentioned technology has broad applications while comprising online remote medical treatment, online conferences, medical education, preparation of surgical plans, etc. Moreover, technical, security, and financial challenges should be tackled down by the future widespread use of metaverse. Metaverse is limitlessly promising, and it will exert a certain effect on future scientific and technological advancements in the medical industry. The review article primarily aims to summarize the application of the metaverse in medicine and their challenge in the future of medicine.

## Introduction

**FIGURE 1:**
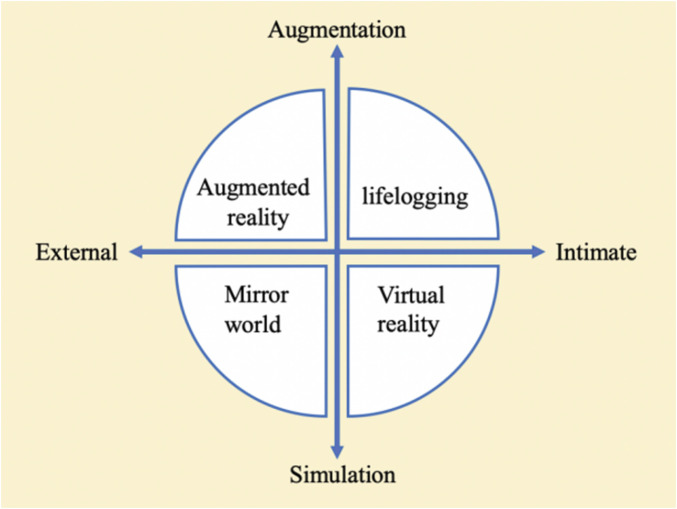
Diagram of 4 types of the metaverse based on 2 axes (Kye et al., 2021).

All persons in practice have a network avatar in 3-dimensional (3D) virtual reality, termed metaverse, which was first proposed by American novelist Neal Stephenson in his novel “Snow Crash” published in 1992. Metaverse has become a reality with advances in technology and society, and it has been extensively known in a wide variety of fields. The Acceleration Studies Foundation (ASF), a reputable metaverse research group, has released the four categories of metaverse in 2006, which were dependent on the two axes (Kye et al., 2021). To be specific, the four categories of metaverse comprise augmented reality (AR), lifelogging, mirror worlds, and virtual reality (VR), the two axes refer to augmentation versus simulation and intimate versus external (Figure 1). The above-mentioned categories and axes can reveal whether the implemental space is reality-centered or virtual-centered, as well as whether the implemental information is external environment information-centered, or intimate information-centered. It is admitted that metaverse is rapidly spreading to all industries. In 2021, metaverse was defined newly as “a world in which virtual and reality interact and co-evolve, and social, economic, and cultural activities take place in them to create value” by Lee (SPRi, 2021). Furthermore, it has been defined as “a 3D-based virtual reality in which daily activities and economic life are conducted through avatars representing the real themselves” by Go (Sparkes, 2023).

### Augmented reality (AR)

Augmented Reality (AR) refers to a virtual three-dimensional (3D) version of reality world over which external information is transformed computer-generated virtual information. The real-time interactive experience between real world and virtual world has been advancing user’s experience of existing real world (Ayoub and Pulijala, 2019). In general, users have employed glasses, lenses, smartphone, and computers, with the typical examples of AR stereoscopic images and 3D construction.

### Virtual reality (VR)

Virtual reality has been recognized as a completely virtual 3D world. In this world, individual information is superimposed on 3D computer generated environments that users are enabled to immerse themselves in for interaction and exploration (Bruno et al., 2022). Users register a virtual personalization avatar in the virtual world for activation and communication. The specific examples cover virtual reality training program and virtual hospitals.

### Lifelogging

Lifelogging refers to a record-keeping platform where users can record their real individual information using a virtual format to share with others, with typical examples of Instagram, Facebook, Twitter, and health monitors (Petrigna and Musumeci, 2022).

### Mirror worlds

Mirror world (e.g., Zoom meeting, BizConf Video, and 360-degree video) is a virtual world that is replicated in accordance with real external events to create a repeated environmental experience for users.

A mirror copy of the real world is termed metaverse, which is generated using a mix of digital science and technology (Davis et al., 2009; Skalidis et al., 2022a). Undoubtedly, metaverse can significantly affect people’s lives as a versatile platform where a considerable number of activities are being conducted, and a critical revolution in medicine may be launched.

The conventional medical model practice is dependent on direct physical contact, whereas it is subjected to the drawbacks of being time-consuming and expensive to get there, whereas technological healthcare has been accelerated by the Corona Virus Disease 2019 (COVID-19). Since 2019, this new reality has completely changed how science and technology are developed in the medical industry, such that it has aroused wide attention from researchers. Metaverse has provided a novel perspective regarding medical development and opened a significant position in the field of health. The above-mentioned technologies are progressively entering the mainstream. Innovative technology has rapidly gained popularity and thrived in the medical and health sectors since digital technology has been leaping forward. Metaverse has been confirmed as one of the revolutionary technologies for its successful and innovative idea. Since metaverse is merging, numerous medical professionals will explore its wide applications in depth. Currently, metaverse has been extensively employed in the medical field.

Thus, insights into the concept and types of the metaverse and examples of its medical applications should be gained.

## Review

### Telemedicine and meeting

Patients can greatly benefit from telemedicine which makes countries’ medical systems unrestricted of time and space especially in rural areas. Numerous virtual hospital apps can offer functionalities of medical consultation, health monitoring, outpatient appointments, even online payment for medical project (Wang et al., 2020). Virtual hospital platform (e.g., spine Metaverse) has proposed the possible application in spine care, with the aim of overcoming several obstacles (e.g., general unaffordability of advanced care, shortages of nursing, hospital beds, and nursing homes) (Chapman et al., 2022). Remote surgery maximizes the use of available medical resources while educating a novel generation of medical professionals who will completely reimagine the medical system (Berber et al., 2016; Veneziano et al., 2020; Zheng et al., 2020; Zhao et al., 2022). The 12-lead electrocardiogram equipment, regardless of the participants’ locations, blood pressure and heart rate monitors, and pulse oximeters are all already available telemedicine services in cardiovascular medicine; the relevant results can be evaluated during the visit (Skalidis et al., 2022a; Skalidis et al., 2022b). In stomatology, metaverse can facilitate remote oral health education or conversations for target participants with no quantitative restriction, which takes on critical significance in the oral health of children in poor mountain areas, and a headset with a see-through eye display 3D images of canal morphology based on CT scans or X-ray version (Albujeer and Khoshnevisan, 2022; Kurian et al., 2022). Telemedicine is applied for disease management and care, as well as for Global Positioning System in patient to get treatment immediately. Telemedicine is capable of significantly reducing the barriers for patients to receive medical treatment, such that doctors are enabled to break through geographical limitations and maximize the utilization of medical resources. Patient data sharing can contribute to the advance of clinical research.

Online conferences include 2D images and 3D environment with digital avatars, we will be able to participate in worldwide events with our coworkers while moving around freely with holographic avatars and exchanging cutting-edge medical knowledge with experts. To train specialists from a wide range of fields in multidisciplinary conversations and examine an imaging series of 2D slices to shared navigation in a 3D organ, the fetal medicine and gynecology fields have employed metaverse (Werner et al., 2022).

### Education and training

**FIGURE 2:**
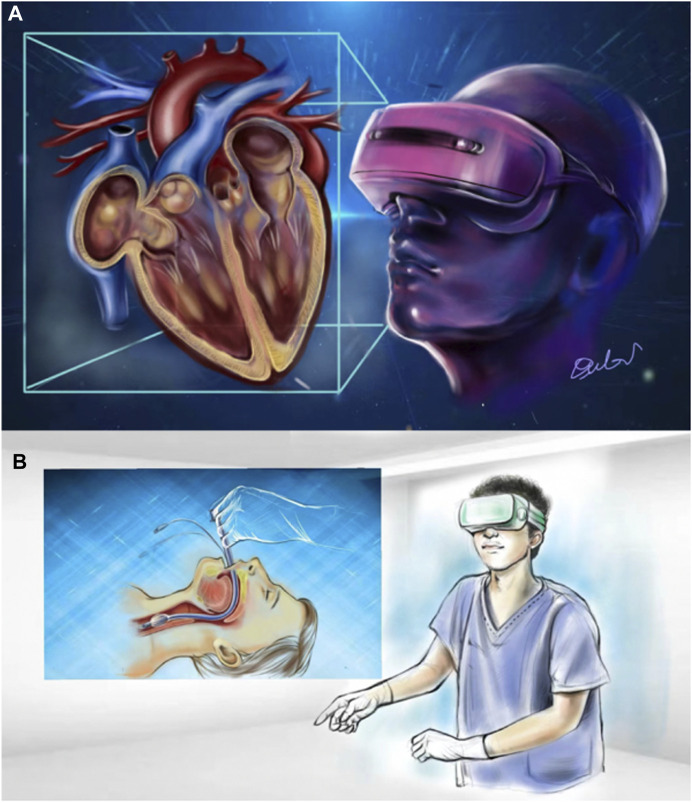
**(A)** Anatomical education through VR; **(B)** A virtual reality endotracheal intubation training (Khundam et al., 2021).

Metaverse can optimally serve as a training platform for medical education. For instance, students can grasp surgical procedures through virtual video using VR glasses (Figure 2A) or software (e.g., Atlas), while they can show 3D mechanism diagrams to visualize biological reactions. Smart operating rooms are adopted to train lung cancer surgery using metaverse extended reality to fulfill the growing need for non-face-to-face education in the Korean medical community (Koo, 2021). A virtual reality training program for endotracheal intubation (Khundam et al., 2021) published in the Journal of educational evaluation for health professionals (JEEHP) (Figure 2B). 360-degree video, the mirror world with scenarios similar to the real environment and avatars that can interact with each other, enable students to learn the basics of emergency care and experience surgical procedures more easily without time restriction (Wu and Ho, 2023). Mirror world may be more promising in the coming years in education of prehospital and disaster medicine. Another realization refers to the haptic gloves applied for dental students who can feel virtual objects while suturing or blocking nerves (Kurian et al., 2022). It was discovered that medical education has improved. A virtual reality medical training system that can learn and train on its own will undoubtedly be created in the future to keep abreast with the advancement of social education today. Seoul National University Bundang Hospital created the platform for spine surgery using augmented reality (MS. P, 2021), and this platform is capable of showing a real-time projection of pedicle screws for spinal fixation on the human body structure. Based on the above-described platform, they will develop a spinal surgery education program to implement an effective education system. Our hospital has a simulated bronchoscope simulator and gastroscope simulator, thus providing young doctors with training and practice before the actual operation for improving the smoothness, success of the medical operation, and the examination experience of patients. As revealed by the above analysis, the healthcare sector is already using VR/AR, i.e., the core technology of the metaverse and will benefit a lot from it in the future. The potential of the metaverse as a novel educational tool is elucidated below: a novel learning environment free from time and location restrictions; a virtual platform allowing students to practice medical procedures repeatedly without worrying about the repercussions of failure; the ability to experience and fully immerse themselves in reality through virtualization.

### Metaverse makes surgery more successful

**FIGURE 3:**
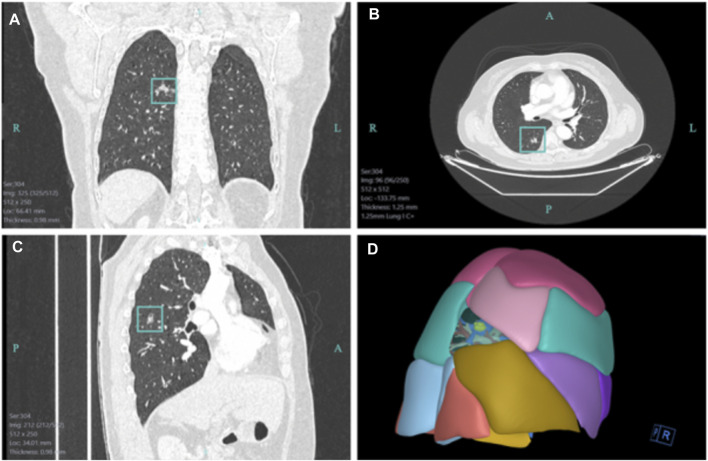
The 3D reconstruction image based on the 2D CT image in the First Hospital of Jilin University. **(A)** Coronal view of a 2D CT lesion. **(B)** Transverse view of a 2D CT lesion. **(C)** Sagittal view of a 2D CT lesion. **(D)** Three-dimensional reconstruction image based on the 2D CT lesion.

The metauniverse-based virtual 3D representation makes the surgery go on more smoothly than before. Volonté et al. (2013) reported the use of AR stereoscopic images of the Da Vinci robot for cholecystectomy. It has also been reported that AR technology provides doctors with a detailed view of important anatomy to guide the successful implementation of endoscopic radical prostatectomy (Chen et al., 2014; Mahmud et al., 2015). AR has been explored in the field of cardiac intervention, AR-based image analysis tools to measure the diameter and length of fluoroscopically identified vessel strictures might facilitate accurate stent selection for optimal treatment (Chen et al., 2014). AR can work with existing endoscopy set-ups makes clear the classification of polyps under gastroscope and improves the detection rate of adenomas (Mahmud et al., 2015). AR smart glasses have been confirmed with tactical advantages in prehospital triage in comparison with conventional approaches during mass casualty incidents (Wu and Ho, 2022). 3D reconstruction, a critical technology for establishing VR to express the objective world on a computer, has been employed in numerous subjects to reconstruct the two-dimensional (2D) CT image into a 3D image to display the lesion location and surrounding important tissues. For thoracic surgeons, 3D reconstruction holds significant clinical value for preoperative assessments regarding lesions and blood vessels (Figure 3). 3D reconstruction helps clinicians develop a more favorable operation plan of patients and increase operation safety (Jackson et al., 2003; Zhang and Liu, 2016; Song et al., 2021). Subsequently, using 3D printing technology, 3D images can be converted into solid objects that are currently applied in orthopedics, congenital heart disease, and thoracic surgery for the chest wall reconstruction and achieved good curative effect and functional reconstruction (Smelt et al., 2020; Wu et al., 2021). With the progress of science and technology, 1 day, the preoperative surgeon can perform surgical operation repeatedly before realistic surgery on the 3D image, such that the actual scene of the lesion can be completely reproduced in metaverse.

### Metaverse for disease management

VR-based Metaverse has served as efficient tools in the treatment of various mental health disorders (Usmani et al., 2022). To be specific, VR cognitive therapy has been employed in delusions, psychosis, and schizophrenia. VR social skills training session allowed anxiety, phobias, and post-traumatic stress disorder patients to engage and acquire skills in communication. Moreover, children who are subjected to attention deficit hyperactivity disorder are more receptive to VR-environment for patient compliance. As revealed by the results, VR is capable of achieving pain management by distracting and stimulating patients to generate neurophysiological changes; it is conducive to reducing opioid use and misuse (Gupta et al., 2017). Notably, it takes on critical significance in pediatric pain and management which is a headache for doctors. Besides, VR can create a scenery environment outside the hospital to facilitate mental health of cancer patients.

### Challenges and developments

**FIGURE 4:**
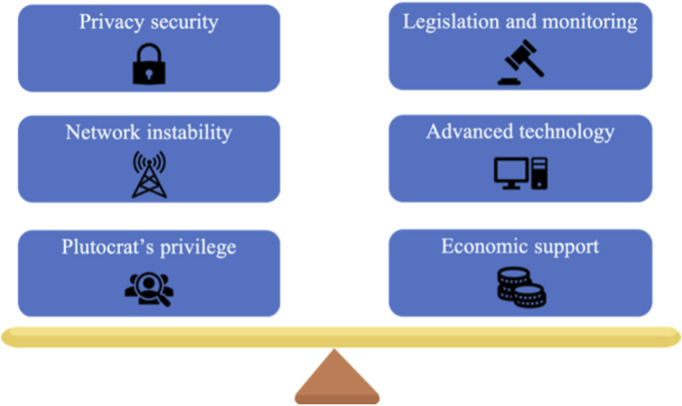
Challenges of a metaverse in medical application.

However, for data leakage and network stability, despite its significant negative effects on patient privacy, network signal disruption, and security, metaverse can exert positive and adverse effects simultaneously (Figure 4). To make it safe for usage, the problems should be addressed. The usage of non-fungible tokens (NFTs), a security measure for patient privacy, which are distinct data units registered in blockchains and adopted to track ownership of digital assets, is likely to be a feasible answer (Bamakan et al., 2022; Skalidis et al., 2022b; Gambril et al., 2022). Advanced science and technology are required to support the widespread use of the metaverse in medicine, comprising a stable and streamlined network, high-quality computer processors, the creation of VR equipment, etc. Nevertheless, advanced science and technology cannot exist independently of economic support. To assure medical safety, considerable clinical trials should be performed for metaverse. In-depth research into cost-saving measures for the widespread use of metaverse in medical care should be further conducted to ensure that it does not merely benefit a small group of wealthy plutocrats. Furthermore, future security efforts will place a high premium on perfecting network security regulations and monitoring. The relevant field will be changed through the application of the metaverse in diagnosis, therapy, medical education, postoperative follow-up, and other areas of medicine (Sandrone, 2022). It is also noteworthy that the development of medical-associated businesses will be more convenient and efficient.

### Negative impacts of metaverse

All things have dualities, and the metaverse can certainly bring about some serious problems. The real world and the virtual world, or the “metaverse,” coexist with contradictions, which manifest in the competition for human energy and limited time. If the metaverse is not used correctly, it can consume a significant amount of people’s time and energy, causing addiction and an inability to extricate themselves, especially among young people. Currently, the negative reports about the metaverse mainly focus on issues related to excessive use, such as physical and mental health problems caused by overuse, and concerns about information security (Ferguson, 2015; Shensa et al., 2017; Amano et al., 2023; Fang et al., 2023). For instance, excessive use of virtual reality devices may lead to adverse symptoms such as eye fatigue, headaches, and dizziness, while long periods of sitting and lack of exercise can also have negative effects on physical health. The long-term virtual reality experiences in the metaverse may conflict with the demands of the real world, leaving individuals feeling confused, anxious, and unsatisfied in their real lives, which can trigger psychological problems. Moreover, the metaverse contains a vast amount of information, but it also harbors a significant amount of false information and inaccurate content. Users may face information overload and have difficulty distinguishing between truth and falsehood (Dong et al., 2023). The security and privacy of users’ personal information and data may also be at risk (Chow et al., 2022). The realization of the Metaverse is envisioned by many as requiring the use of visualization technologies such as VR and AR. This visual aspect of the Metaverse will undoubtedly give rise to emerging cybersecurity threats that have not received much attention. In clinical applications, the development of high-quality clinical work such as remote diagnosis, treatment, and remote robotic surgery through the metaverse is hindered by the metaverse’s high dependence on the speed and stability of networks. What needs to be noted is that these are potential risks and issues, and their specific impact still depends on the actual operation of the metaverse and individual usage methods. With the ongoing development of 5G technology and stronger regulation, it is hopeful that these adverse impacts can be addressed and mitigated, thus ensuring the health and sustainable development of the metaverse (Marescaux et al., 2002; Moustris et al., 2023).

## Conclusion

In this study, we aim to delve into the applications and advantages of the metaverse in the medical and health fields while also exploring its potential negative impacts. The Metaverse is currently being hailed as the next major technological disruption that could have a significant impact on clinician-patient interactions, patient experience, and the processes of innovation and research and development. The revolution of the metaverse is already underway, making it essential for clinicians to embrace it as protagonists in order to guide it in the right direction.
